# Increased rate of multidrug-resistant gram-negative bacterial infections in hospitalized immunocompromised pediatric patients

**DOI:** 10.3389/fcimb.2024.1382500

**Published:** 2025-01-06

**Authors:** Sarah Khafaja, Yara Salameh, Celina F. Boutros, Cherine Awad, Kawthar Faour, Nadim Tfaily, Sarah Merhi, Zeinab El Zein, Samer Bou Karroum, Dana Oweini, Danielle Fayad, George F. Araj, Ramia Zakhour, Ghassan S. Dbaibo

**Affiliations:** ^1^ Center for Infectious Diseases Research (CIDR) and WHO Collaborating Center for Reference and Research on Bacterial Pathogens, American University of Beirut, Beirut, Lebanon; ^2^ Department of Pediatrics and Adolescent Medicine, American University of Beirut Medical Center, Beirut, Lebanon; ^3^ Clinical Microbiology Laboratory, American University of Beirut Medical Center, Beirut, Lebanon; ^4^ Department of Pathology and Laboratory Medicine, American University of Beirut Medical Center, Beirut, Lebanon

**Keywords:** multidrug resistance, gram-negative bacteria, antimicrobial resistance, immunocompromised, children, adolescent, cancer, inborn errors of immunity

## Abstract

**Introduction:**

Multidrug resistant Gram-negative bacterial infections are considered a major public health threat. Immunocompromised pediatric patients are at a great risk of severe or overwhelming infections. The aim of this study was to describe the frequency of infections with multidrug resistant (MDR) Gram-negative bacteria (GNB) in immunocompromised pediatric patients and to determine the risk factors. In addition, we aimed to identify the antimicrobial resistance patterns of these isolates.

**Materials and methods:**

This was a retrospective observational study conducted at the American University of Beirut Medical Center (AUBMC) from 2009 to 2017. The study included immunocompromised patients 18 years of age or younger with infections caused by Gram-negative bacteria isolated from a sterile site, or nonsterile site in the setting of clinical infection.

**Results:**

A total of 381 episodes of infection with GNB in 242 immunocompromised pediatric patients were identified. The mean age was 7.7 years. The most common pathogens were *Enterobacterales* followed by *Pseudomonas* and *Acinetobacter* spp. MDR GNB infections predominated causing 72% of the episodes, with alarming MDR rates among *Escherichia coli* (95.7%) and *Klebsiella pneumoniae* (82.7%). The overall rate of MDR GNB isolated increased from 62.7% in 2015 to 90% in 2017. Thrombocytopenia, chemotherapy and previous colonization or infection with the same organism during the past 12 months were found to be independent risk factors for infection with MDR GNB.

**Conclusion:**

This study provides data on the epidemiology of infections with MDR GNB in immunocompromised pediatric patients and illustrates the alarmingly high prevalence of these infections. This necessitates the frequent revisiting of treatment guidelines in these high-risk patients and the implementation of judicious antimicrobial stewardship programs and infection control policies to stabilize or decrease the prevalence of these infections.

## Introduction

Antimicrobial resistance (AMR) has emerged as a significant global health threat of the 21^st^ century, associated with poor outcomes, increased mortality, healthcare-associated infections, length of hospital stay and economic costs, requiring urgent containment measures (WHO, 2021a; Murray et al., 2022). The World Bank, in the 2017 report “Drug-Resistant infections: a threat to our economic future” describing the destructive impacts of AMR on the global economy from 2017 through 2050, estimated that by 2050, AMR can cause a global increase in healthcare costs ranging from $300 billion to more than $1 trillion per year, a 1.1% to 3.8% reduction in the annual global gross domestic product and can push between 8 to 28 million people into extreme poverty (Bank TW, 2017; National Academies of Sciences E, and Medicine, 2022).

Among the numerous challenges of AMR, the emergence and rapid spread of multidrug resistant Gram-negative bacteria (MDR GNB) in particular, poses a serious threat to public health and healthcare systems, especially that treatment options in the pediatric population, are rapidly declining leading to significant increase in morbidity and mortality (Karaiskos et al., 2019; Alshammari, 2021; WHO, 2021b; WHO, 2023). According to the antibiotic resistance threats report by the Centers for Disease Control and Prevention (CDC) updated in 2019, extended spectrum beta-lactamase (ESBL)-producing *Enterobacterales*, drug-resistant *Campylobacter*, MDR *Pseudomonas aeruginosa*, drug-resistant nontyphoidal *Salmonella*, drug-resistant *Salmonella* serotype typhi, drug-resistant *Shigella* were classified as serious threats, whereas carbapenem-resistant (CR) *Acinetobacter*, carbapenem-resistant *Enterobacterales*, drug-resistant *Neisseria gonorrheae* were considered urgent threats (CDC, 2019).

MDR is defined as acquired non-susceptibility to at least one agent in three or more antimicrobial categories (Magiorakos et al., 2012). MDR GNB are particularly prevalent among immunocompromised patients who are more susceptible to septic complications, overwhelming infections, and poor outcomes leading to widespread use of antibiotic prophylaxis and empirical therapy with activity against GNB in this population (Montassier et al., 2013; Hernández-Jiménez et al., 2022). A retrospective study conducted from 2007 to 2018 in Brazil showed that immunocompromised patients had an 8.5-fold higher chance of MDR bacterial infection when compared to non-immunocompromised patients (Silva et al., 2022). *Enterobacterales* cause 65%–80% of documented Gram-negative infections in cancer patients (Tohamy et al., 2018). In recent years, a change in the epidemiology of bacteremia was reported, with a shift from Gram-positive to Gram-negative bacteria among bacterial infections in patients with cancer (Montassier et al., 2013). Several factors have been implicated, including the use and duration of antibiotic prophylaxis, the nature of chemotherapy (myeloablative or non-myeloablative) or the reduction in the use of indwelling catheters leading to a decrease in the incidence of catheter-related bacteremia, mostly due to Gram-positive organisms (Kanj and Kanafani, 2011; Montassier et al., 2013). Infections with MDR GNB develop through 5cross-contamination and antibiotic pressure, therefore immunocompromised patients are at high risk of developing infections with MDR GNB, due to multiple factors including prolonged periods of neutropenia, hematopoietic stem cell transplantation, frequent and/or long exposure to antibiotics, and aggressive treatment such as chemotherapy, radiation therapy or steroids, which impair both innate and adaptive immune systems and gut microbiota eubiosis (El-Mahallawy et al., 2011a; Costa et al., 2015; Baker and Satlin, 2016; Alagna et al., 2020; Russell et al., 2023).

Little is known regarding the risk factors and outcomes of infections with MDR GNB in immunocompromised patients in the Middle East and North Africa (MENA) region (Al Dabbagh et al., 2023). The scarcity of epidemiological data in this vulnerable population makes it difficult to estimate the true burden of the problem. Broadening our knowledge of the local prevalence of MDR GNB and their resistance patterns, as well as the identification of risk factors for infection with MDR bacteria are fundamental steps to improve treatment protocols and the choice of empiric antibiotic therapy (Montassier et al., 2013; Trecarichi et al., 2015a). For this purpose, the aim of this study was to describe the epidemiology, risk factors, and antimicrobial resistance pattern for infections with MDR GNB.

## Materials and methods

This was a retrospective medical record review of children and adolescents aged 18 years or younger, admitted to the hospital with Gram-negative bacterial infections, from June 1^st^, 2009, to June 31^st^, 2017. The study was conducted at the American University of Beirut Medical Center (AUBMC), a tertiary care medical center located in Beirut, Lebanon, with a total of 420 hospital beds and around 9500 pediatric inpatient admissions annually serving as a referral center for pediatric patients with cancer or immunodeficiency. Also, AUBMC hosts the regional non-profit Children’s Cancer Center of Lebanon (CCCL) affiliated with the St. Jude’s Children’s Research Hospital in Memphis, Tennessee, USA. In addition, patients with inborn errors of immunity (IEI) are frequently referred to the Primary Immunodeficiency Diseases Program at AUBMC. This study was approved by the institutional review board (IRB) at AUBMC (IRB ID: BIO-2019-0019).

A list of all positive cultures yielding Gram-negative bacteria (GNB) was obtained through the medical records department. The medical records of patients with positive cultures were reviewed based on the below inclusion and exclusion criteria.

### Inclusion and exclusion criteria

Inclusion criteria were: 1- Positive cultures for Gram-negative bacteria collected from a sterile site or nonsterile site in the setting of clinical infection such as tracheal aspirate culture in the presence of clinical and radiographic findings suggestive of pneumonia, wound culture in the setting of surgical wound infection, or others; 2- Cultures taken from immunocompromised patients 18 years of age or younger admitted to the hospital during the study period.

To avoid duplication of isolates, cultures obtained on the same day or on different dates but responsible for the same episode of infection were excluded with only the first culture from each episode being included, unless it was collected from different sites, or it yielded different organisms or same organisms with different antimicrobial resistance pattern. When different organisms were identified from different sites during the same episode, they were treated as separate episodes.

Exclusion criteria were: 1- Positive culture for GNB determined to reflect colonization rather than true infection by the treating physician; 2-Incomplete charts; 3-Episodes of infection with GNB in outpatients.

### Definitions and categorization

Categorization of isolates into MDR and non-MDR GNB was based on a standardized international terminology, created by a group of international experts that came together through a joint initiative by the European Centre for Disease Prevention and Control (ECDC) and the CDC, who described the acquired resistance profiles in *Enterobacterales* (other than *Salmonella* and *Shigella*), *Pseudomonas aeruginosa* and *Acinetobacter* species (*spp.)* MDR was defined as acquired non-susceptibility to at least one agent in three or more antimicrobial categories (Magiorakos et al., 2012). The characterization of other organisms, not included in their definition, was carried out as previously reported by our Clinical Microbiology Laboratory, a College of American Pathologists-accredited laboratory since 2004 (CLSI, 2023). *Stenotrophomonas maltophilia* is considered an intrinsically MDR organism (Brooke, 2012).

*Enterobacterales* refer to: *Escherichia coli, Shigella* spp. (*Shigella flexneri, Shigella sonnei*)*, Klebsiella* spp. (*Klebsiella oxytoca*, *Klebsiella pneumoniae*), *Proteus mirabilis*, *Citrobacter* spp. (*Citrobacter koseri, Citrobacter freundii)*, *Serratia* spp. (*Serratia marcescens, Serratia liquefaciens)*, *Morganella morganii*, *Enterobacter* spp. (*Enterobacter aerogenes*, *Enterobacter cloacae*, *Enterobacter agglomerans*), *Salmonella* spp. (*Salmonella enteritidis*, *Salmonella* group C, *Salmonella paratyphi*, *Salmonella* not typable).

*Pseudomonas* spp. refer to: *Pseudomonas aeruginosa*, *Pseudomonas fluorescens*, *Pseudomonas putrefaciens*, *Pseudomonas stutzeri*, *Pseudomonas* spp.

*Acinetobacter* spp. refer to: *Acinetobacter anitratus*, *Acinetobacter baumanii*, *Acinetobacter lwoffi*.

Immunocompromised status was defined as those patients with hematological malignancies or solid tumors (active or in remission for less than 5 years), IEI, or those receiving long term (>30 days) or high dose (> 1 mg per kilogram per day) steroids or other immunosuppressive drugs (Lemiale et al., 2015; Russell et al., 2023).

Infections, as opposed to colonization (asymptomatic carrier state), were defined by clinical, biological and imaging characteristics according to the definitions published by international societies on community or healthcare-associated pneumonia (Bradley et al., 2011) bloodstream and catheter related infections (Mermel et al., 2009), urinary tract infections (Mattoo et al., 2021), and other community- and healthcare-associated infections (Solomkin et al., 2010; Kreitmann et al., 2023).

Regarding the therapeutic strategy, monotherapy was defined as the use of one antibiotic, whereas combination therapy was defined as the concomitant use of at least two antibiotics for more than 48 hours.

### Bacterial isolates, identification, and antimicrobial susceptibility testing

Isolates of GNB recovered from clinical samples submitted to the Clinical Microbiology Laboratory, Department of Pathology and Laboratory Medicine, AUBMC, were identified based on standard biochemical methods or using the matrix-assisted laser desorption/ionization time of flight (MALDI-TOF) system (Bruker Daltonik, GmbH, Bremen, Germany) starting 2015. The antimicrobial susceptibility testing was performed using the disk diffusion test and interpreted according to the Clinical and Laboratory Standards Institute (CLSI) guidelines published for each year. The minimal inhibitory concentrations (MICs) of carbapenems, colistin and tigecycline were determined when requested by the medical team, using the E-test (PDM-Epsilometer, AB Biodisk, Solna, Sweden) according to the manufacturer’s guidelines.

### Data collection

Data was collected using a case report form, which included the following information: basic demographic and epidemiological characteristics (age, binary sex, nationality, and residence), type of immunocompromising condition, medical comorbidities, and placement of invasive device or undergoing invasive procedure in the 30 days prior to infection. Presence of neutropenia or thrombocytopenia, previous hospital or pediatric intensive care unit (PICU) admission, surgical intervention or antibiotic use within the previous 12 months were also recorded, as well as the history of previous infection or colonization with the same organism or any other MDR GNB. In addition, management and outcomes including length of hospital stay, PICU admission, the need for mechanical ventilation, recurrence of infection or resolution and mortality were recorded. Microbiological data including isolated organisms and antimicrobial resistance profiles were also collected.

### Statistical analysis

The Statistical Package for Social Sciences (SPSS) program, version 25.0 for Windows was used for data analysis (IBM, Armonk, NY). Simple descriptive statistics was used to describe patients’ demographics, organisms’ distribution and antimicrobial resistance. Bivariate and multivariate analyses of risk factors for MDR GNB infection was analyzed by Pearson’s Chi-square test or Fisher’s exact test (when the number of patients in a subgroup was less than 5). Continuous risk factors were analyzed with student *t* test. Statistical significance was considered below a type-1 error threshold (alpha level) of 0.05. Odds ratio (OR) was reported to compare the relative odds of having MDR infection given a particular condition compared to the odd of MDR infection in the absence of this condition. Following that a multivariable logistic regression model comprised of all risk factors for MDR infections with unadjusted *p*-value < 0.2 was subsequently constructed to account for the different confounding factors. Adjusted odds ratios (ORa) were then reported.

## Results

**Figure 1:**
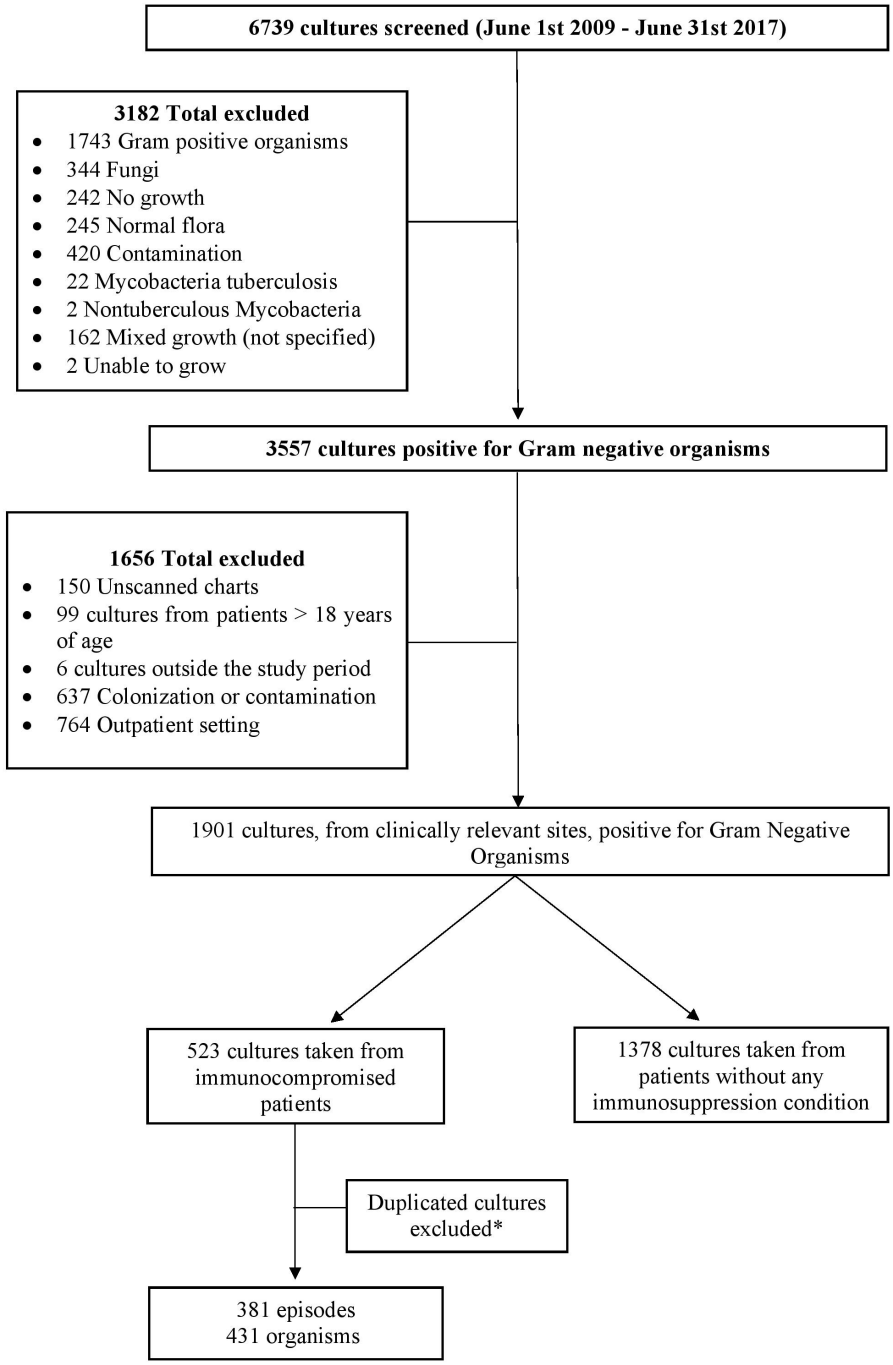
Flow chart of the study design. *Duplicated cultures, from same or different dates, responsible for the same episode of infection were excluded and only the first culture from each episode was included unless it was collected from different sites. Different cultures taken from the same site, during the same episode of infection, that yielded different organisms were included.

In the 8-year study period, a total of 6739 cultures were screened, of which 1901 cultures collected from clinically relevant sites were positive for GNB. A total of 523 cultures were identified from immunocompromised patients. After removal of the duplicated cultures, the remaining cultures accounted for 381 episodes in 242 patients with immunosuppression, and a total of 431 organisms were documented (**Figure 1**). Based on the definition by the joint initiative by the ECDC and the CDC, 72% of the episodes were MDR.

**Supplementary Figures S1** and **S2** show the incidence of MDR gram-negative isolates per 100,000 immunocompromised pediatric patients, as well as the incidence rate ratios (IRRs) across the years. We observe that the incidence of MDR gram-negative isolates peaked in 2010, followed by a gradual decline. Additionally, the IRR was nearly 2.5 between 2009 and 2010, then decreased substantially to 0.2 between 2010 and 2011. From 2011 to 2016, the IRR fluctuated between 0.8 and 1, before decreasing again to 0.4 between 2016 and 2017.

### Socio-demographic, clinical characteristics, and risk factors

**Table 1** summarizes the sociodemographic characteristics. Females were more likely to have MDR GNB infections compared to males. Otherwise, both groups were similar in terms of age, residence, nationality, and underlying comorbidities.

**Table 1:** Comparison of demographics and baseline characteristics of patients with Gram-negative bacterial infections caused by multidrug resistant or non-multidrug resistant isolates.

	Non-MDR (N=107)	MDR (N= 274)	p-value
**Age, years (mean ±SD)**	7.7 (± 5.8)	7.3 (± 5.7)	0.569
**Binary sex, n (%)**			**0.026**
Male	44 (41.1)	80 (29.2)	
Female	63 (58.9)	194 (70.8)	
**Nationality**			0.504
Lebanese	88 (82.2)	217 (79.2)	
Non-Lebanese	19 (17.8)	57 (20.8)	
**Residence**			0.106*
Beirut	15 (14.0)	41 (14.2)	
Mount Lebanon	41 (38.3)	72 (26.3)	
South Lebanon	13 (12.1)	61 (22.3)	
North Lebanon	15 (14.0)	52 (19.0)	
BeKaa	10 (9.3)	22 (8.0)	
Baalbeck-Hermel	2 (1.9)	1 (0.4)	
Nabatiyeh	0 (0.0)	1 (0.4)	
Akkar	0 (0.0)	1 (0.4)	
Outside Lebanon	11 (10.3)	25 (9.1)	
**Underlying comorbidities**	17 (15.9)	30 (10.9)	0.188
Cardiovascular diseases	5 (4.7)	7 (2.6)	0.330*
Pulmonary diseases	3 (2.8)	7 (2.6)	1.000*
Neurologic diseases	9 (8.4)	14 (5.1)	0.224
Renal diseases	2 (1.9)	5 (1.8)	1.000*
Gastrointestinal diseases	3 (2.8)	4 (1.5)	0.406*
Other chronic diseases+	4 (3.7)	3 (1.1)	0.101*

Pearson’s Chi-Square test was used (no expected count less than 5).

*Fisher’s exact test was used when expected count was less than 5.

+Other chronic diseases: Takayasu arteritis, systemic lupus erythematosus and aplastic anemia. The bold values refer to the significant factors that have a p-value <0.05.

**Table 2** shows the different immunocompromised states that were identified. The main types were hematological malignancies (50.9%), followed by solid tumors (34.1%), IEI (11%) and receipt of immunosuppressive drugs (3.9%), and there were no significant differences between the two groups except that patients with acute myeloid leukemia had higher odds of getting an infection with MDR GNB compared to those with acute lymphocytic leukemia (OR 2.749, CI [1.071-7.053], p-value 0.035).

**Table 2:** The different immunocompromised states encountered in patients with Gram-negative bacterial infections caused by multidrug resistant or non-multidrug resistant isolates.

Type of immunosuppression	Total (N= 381)	Non-MDR (N=107)	MDR (N=274)	p-value^¶^	p-value^§^ (within subgroups)
**Inborn errors of immunity (IEI)**	**42 (11.0)**	**16 (15.0)**	**26 (9.5)**	0.141	
SCID*	11 (26.2)	4 (25.0)	7 (26.9)		Ref
DiGeorge syndrome	7 (16.7)	2 (12.5)	5 (19.2)		0.733
Immunodeficiency unspecified	17 (40.5)	7 (43.8)	10 (38.5)		0.799
Other IEI**	7 (16.7)	3 (18.8)	4 (15.4)		0.783
**Hematological malignancies**	**194 (50.9)**	**50 (46.7)**	**144 (52.6)**	0.939	
Acute lymphocytic leukemia	122 (62.9)	35 (70.0)	87 (60.4)		Ref
Acute myeloid leukemia	47 (24.2)	6 (12.0)	41 (28.5)		**0.035**
Lymphoma	24 (12.4)	9 (18.0)	15 (10.4)		0.392
Natural killer cell leukemia	1 (0.5)	0 (0.0)	1 (0.7)		1.000
**Solid tumors**	**130 (34.1)**	**34 (31.8)**	**96 (35.0)**	Ref	
Bone tumors	35 (26.9)	7 (20.6)	28 (29.2)		Ref
Brain tumors	32 (24.6)	13 (38.2)	19 (19.8)		1.000
Neuroblastomas	21 (16.2)	6 (17.6)	15 (15.6)		0.173
Rhabdomyosarcoma	15 (11.5)	3 (8.8)	12 (12.5)		0.626
Germ cell tumors	15 (11.5)	2 (5.9)	13 (13.5)		0.757
Other solid tumors^+^	12 (9.2)	3 (8.8)	9 (9.4)		0.560
**Immunosuppressive drugs**	**15 (3.9)**	**7 (6.5)**	**8 (2.9)**	0.103	

*SCID: Severe combined immunodeficiency.

**Other IEI: IgA deficiency, X-linked agammaglobulinemia, osteopetrosis, hyper-IgE syndrome.

^+^Other solid tumors: hepatoblastoma, Wilm’s tumor, clear cell sarcoma of the kidney, adrenocortical carcinoma, retinoblastoma, malignant peripheral sheath nerve tumor

^¶^The comparison was performed between the groups, types of immunocompromised state, inborn errors of immunity (IEI), hematological malignancies, solid tumors, and immunosuppressive drugs. Solid tumors were the reference group. All types of immunocompromised state did not show any significant statistical difference between the groups.

^§^The comparison was performed between the subgroups of each group. The odds of having an MDR infection was 2.749 times greater (CI [1.071-7.053]) for patients with acute myeloid leukemia when compared to those with acute lymphocytic leukemia (p-value= 0.035).

Ref: The reference group. The bold values refer to the significant factors that have a p-value <0.05.

There were no significant differences regarding previous hospital or PICU admission, previous surgical intervention, or previous antibiotic use within the past 12 months (**Table 3**). However, the receipt of antibiotic therapy in the past 30 days was significantly more common in the MDR GNB group (68.6% vs 54.2%, p-value 0.008). The MDR GNB group had a higher frequency of previous infection or colonization with the same organism (37.7% vs 17.5%, p-value < 0.001). Among the main risk factors for MDR GNB episodes were neutropenia and thrombocytopenia at admission (**Table 3**). In the multivariable logistic regression, thrombocytopenia (ORa 2.14, 95% CI 1.3-3.5), chemotherapy (ORa 2.12, 95% CI 1.2-3.6) and previous infection or colonization with the same organism (ORa 2.14, 95% CI 1.7-5.4) emerged as independent risk factors (**Table 4**).

**Table 3:** Risk factors for acquiring a multidrug resistant Gram-negative bacterial infection in immunocompromised pediatric patients.

	Non-MDR (N=107)	MDR^+^ (N= 274)	p-value
Transplantation	13 (12.1)	31 (11.3)	0.819
Previous hospitalization in the past 12 months	93 (86.9)	246 (89.8)	0.422
Previous ICU admission in the past 12 months	22 (20.6)	44 (16.1)	0.297
Antibiotics use in the past 12 months	67 (62.6)	187 (71.1)	0.111
Antibiotics use in the past 30 days	58 (54.2)	186 (68.6)	**0.008**
Antibiotic prophylaxis	66 (61.7)	208 (75.9)	**0.005**
Chemotherapy	67 (62.6)	217 (79.2)	**<0.001**
Time between the last chemotherapy and admission, mean ( ±SD)*	9.3 (±11.5)	12.4 ( ±13.8)	0.098
Steroids	38 (35.5)	98 (35.8)	1.000
Surgical intervention in the past 12 months	65 (60.7)	174 (63.5)	0.617
Transfusion of blood products in last 7 days	14 (13.1)	53 (19.3)	0.149
Invasive device in the past 30 days	88 (82.2)	216 (78.8)	0.456
Central line	79 (73.8)	198 (72.3)	0.757
Urinary catheter	14 (13.1)	16 (5.8)	**0.018**
Nasogastric tube	19 (17.8)	24 (8.8)	**0.013**
Invasive prosthesis	7 (6.5)	14 (5.1)	0.582
Invasive procedure in the past 30 days	17 (15.9)	39 (14.2)	0.682
Lumbar puncture	7 (6.5)	23 (8.4)	0.546
Thoracocenthesis	1 (0.9)	3 (1.1)	1.000*
Mechanical ventilation/Intubation	10 (9.3)	13 (4.7)	0.09
Cardiac catheterization	0 (0.0)	1 (0.4)	1.000*
Neutropenia on admision	32 (29.9)	128 (46.7)	**0.003**
Thrombocytopenia on admission	37 (34.6)	151 (55.1)	**<0.001**
Previous infection or colonization with the same organism (N=363), n/N (%)	18/103 (17.5)	98/260 (37.7)	**<0.001**
Previous infection or colonization with the same organism and same resistance to antibiotics, (N=359) n/N (%)	14/103 (13.6)	44/256 (17.2)	0.402
Previous infection with other MDR organism, (N=361) n/N (%)	25/103 (24.3)	69/26.7 (26.7)	0.629

Pearson’s Chi-Square test was used (no expected count less than 5).

*Fisher’s exact test was used when expected count was less than 5.

^+^Four subjects had missing data from the MDR group. The bold values refer to the significant factors that have a p-value <0.05.

**Table 4:** Independent risk factors for infection with multidrug resistant Gram-negative bacteria using multivariable logistic regression.

Risk factors	ORa [95% CI]	p-value
Thrombocytopenia	2.14 [1.3-3.5]	0.003
Chemotherapy	2.12 [1.2-3.6]	0.007
Previous infection or colonization with same organism	3.0 [1.7-5.4]	<0.001

ORa, Adjusted Odds Ratio; CI, Confidence Interval; MDR, Multi-Drug Resistant; GNB, Gram-Negative Bacteria

This multivariate logistic regression included all variables and confounding factors that had a value of p ≤ 0.20 in the univariate analysis, adjusting for age and gender. The method of selection of the variables which has been chosen here is the stepwise method. The reference category is the first category of each factor.

Classification table: Global percentage 73%, Model fit test <0.001, Nagelkerke R-Square 0.141, Goodness of fit test 0.943. The bold values refer to the significant factors that have a p-value <0.05.

### Management and outcome

No differences were observed between MDR GNB and non-MDR GNB groups in the use of combination antimicrobial therapy (**Table 5**). Third and fourth generation cephalosporins were used more often in the non-MDR GNB group (p-value < 0.001 for both) whereas carbapenems and aminoglycosides were more commonly used in the MDR GNB group (p-value < 0.001 and 0.036, respectively). The most common antimicrobial categories used for treatment of MDR GNB were carbapenems (69%) and aminoglycosides (56.3%) (**Table 5**; **Supplementary Figure S3**). There was a significant increase in the carbapenem use from 2009 (37.5%) to 2011 (79.3%) (p-value 0.007), followed by a relatively steady usage between 2011 and 2017. The increase in use of carbapenems coincides with a decrease in use of aminoglycosides, third and fourth generation cephalosporins. Furthermore, the use of aminoglycosides started to increase in 2012 onwards (**Supplementary Figure S3**).

**Table 5:** Management and outcome.

	Total (N=374), n (%)	Non-MDR (N=106), n (%)	MDR (N=268), n (%)	p-value
Combination vs monotherapy				0.291
Antimicrobial monotherapy	224 (59.9)	68 (64.2)	156 (58.2)	
Combination antimicrobial therapy	150 (40.1)	38 (35.8)	112 (41.8)	
Antimicrobial therapy (N=374)				
Third generation cephalosporins	51 (13.6)	28 (26.4)	23 (8.6)	**<0.001**
Fourth generation cephalosporins	75 (20.1)	37 (34.9)	38 (14.2)	**<0.001**
Carbapenems	229 (61.2)	44 (41.5)	185 (69.0)	**<0.001**
Aminoglycosides	198 (52.9)	47 (44.3)	151 (56.3)	**0.036**
Fluoroquinolones	25 (6.7)	12 (11.3)	13 (4.9)	**0.024**
Polymyxins	10 (2.7)	1 (0.9)	9 (3.4)	0.192
Antipseudomonal penicillins with β-lactamase inhibitors	17 (4.5)	4 (3.8)	13 (4.9)	0.788*

	Total (N=381), n (%)	Non-MDR (N=107), n (%)	MDR (N=274), n (%)	p-value
Duration of hospital stay, mean (± SD)^+^	13.8 (10.8)	12.0 (9.2)	14.4 (11.2)	0.073
ICU admission	61 (16.0)	19 (17.8)	42 (15.3	0.561
Need for mechanical ventilation	26 (6.8)	10 (9.3)	16 (5.8)	0.223
Outcome				0.587*
Resolution without sequelae	238 (62.5)	70 (65.4)	168 (61.3)	
Resolution with sequelae	39 (10.2)	10 (9.3)	29 (10.6)	
Recurrence of infection	78 (20.5)	23 (21.5)	55 (20.1)	
Death related to infection	6 (1.6)	0 (0.0)	6 (2.2)	
Death unrelated to infection	20 (5.2)	4 (3.7)	16 (5.8)	

Pearson’s Chi-Square test was used (no expected count less than 5).

*Fisher’s exact test was used when expected count was less than 5.

^+^Only the first episode was included in this analysis if multiple episodes occurred during the same admission. The independent T-test was conducted twice with outliers and without outliers, and the p-values were nearly identical. The numbers represented in the table are based on the analysis after outliers’ removal. The bold values refer to the significant factors that have a p-value <0.05.

The average length of hospital stay for patients with GNB infections was 10 days and there was no significant difference in the length of hospitalization, resolution of infection with or without sequelae, recurrence of infection, or mortality between patients infected with MDR or non-MDR GNB (**Table 5**).

### Microbiology and antibiotic susceptibility

**Figure 2:**
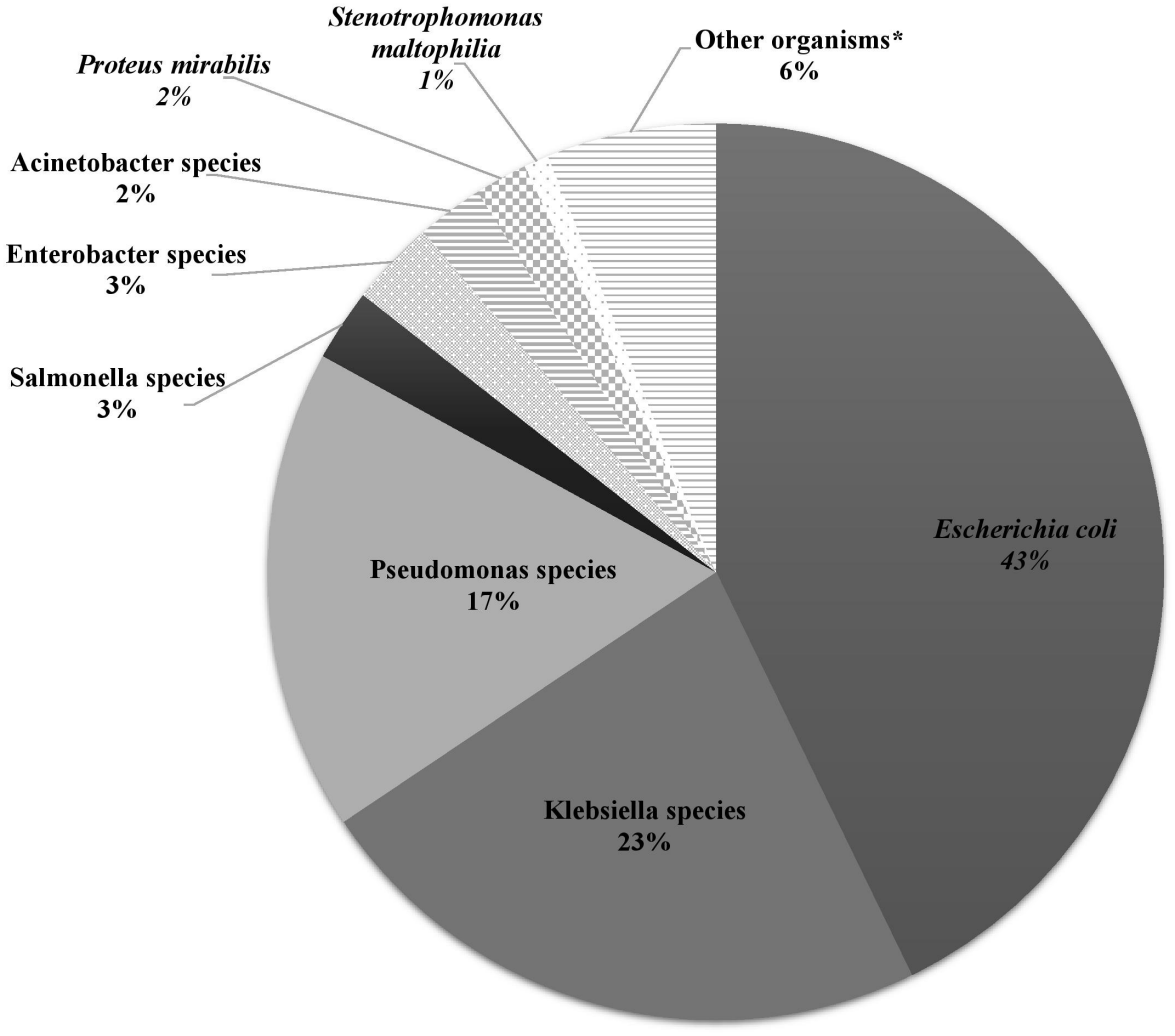
The distribution of Gram-negative bacteria isolated from infected immunocompromised pediatric patients (N =431). *Klebsiella* spp.: *Klebsiella oxytoca* (3), *Klebsiella pneumoniae* (95). *Pseudomonas* spp.: *Pseudomonas aeruginosa* (64), *Pseudomonas fluorescens* (3), *Pseudomonas putrefaciens* (1), *Pseudomonas stutzeri* (6), *Pseudomonas* spp. (1). *Salmonella* spp.: *Salmonella enteritidis* (7), *Salmonella* group C (2), *Salmonella paratyphi* (1), *Salmonella* not typable (1). *Enterobacter* spp.: *Enterobacter aerogenes* (1), *Enterobacter cloacae* (11), *Enterobacter agglomerans* (1). *Acinetobacter* spp.: *Acinetobacter anitratus* (4), *Acinetobacter baumanii* (5), *Acinetobacter lwoffi* (1). *Other organisms: *Aeromonas hydrophilia*, *Aeromonas sobria*, *Brevundimonas vesicularis*, *Campylobacter* spp. (2), *Chryseomonas indologens* (2), *Citrobacter freundii* (3), *Citrobacter koseri*, *Comamonas acidovorans*, *Haemophilus influenzae* not type B, *Moraxella catarrhalis* (2), *Morganella morganii* (2), *Myroides* spp., *Ochobactrum anthropi*, *Ralstonia piketti* (2), *Serratia liquefaciens* (2), *Serratia marcescens*, *Shigella flexneri* (2), *Shigella sonnei*.

**Figure 3:**
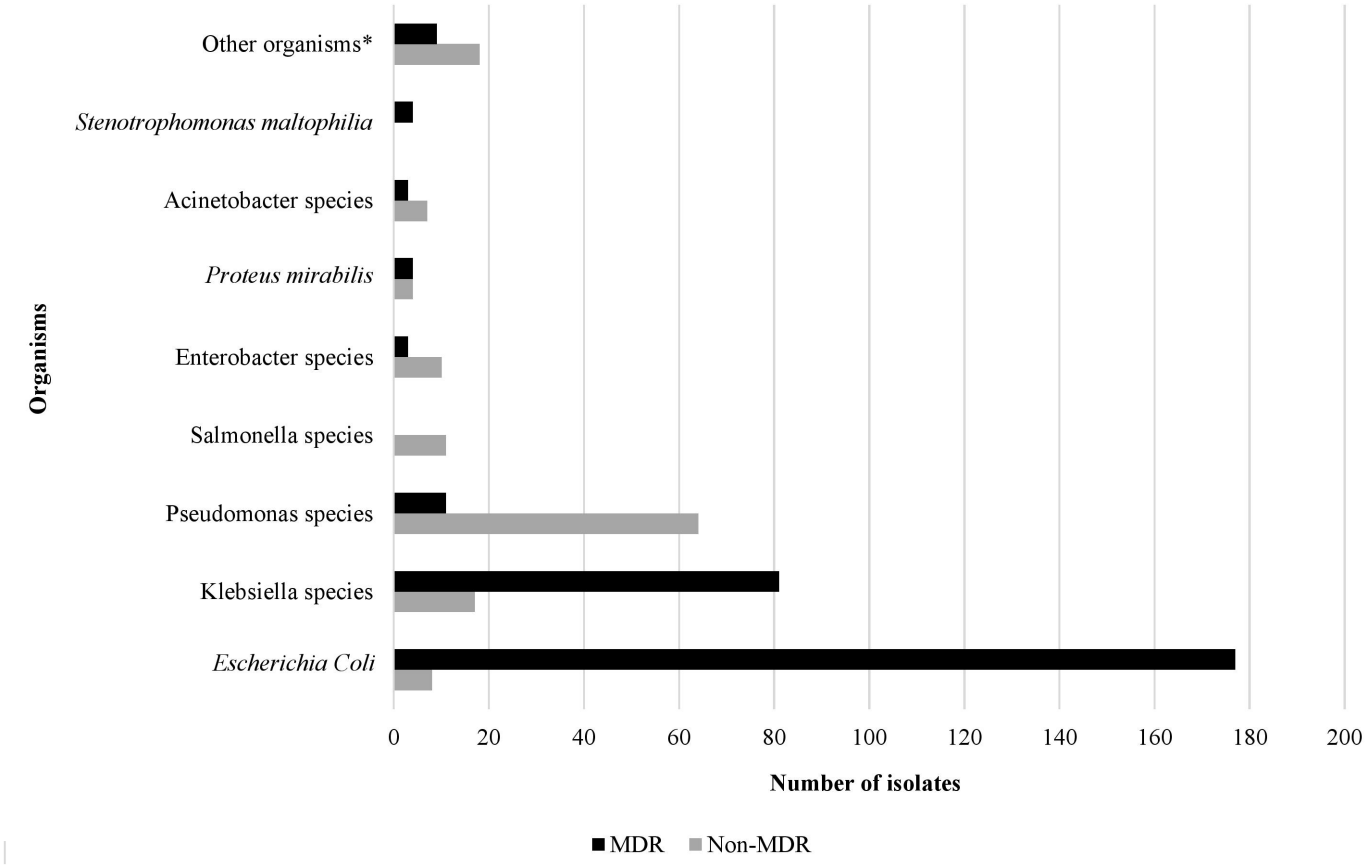
The rate of the multidrug resistant phenotype in Gram-negative bacteria isolated from infected immunocompromised pediatric patients (N=431). *Other organisms: *Aeromonas hydrophilia*, *Aeromonas sobria*, *Brevundimonas vesicularis*, *Campylobacter* spp. (2), *Chryseomonas indologens* (2), *Citrobacter freundii* (3), *Citrobacter koseri*, *Comamonas acidovorans*, *Haemophilus influenzae* not type B, *Moraxella catarrhalis* (2), *Morganella morganii* (2), *Myroides* spp., *Ochobactrum anthropi*, *Ralstonia piketti* (2), *Serratia liquefaciens* (2), *Serratia marcescens*, *Shigella flexneri* (2), *Shigella sonnei*.

**Figure 4:**
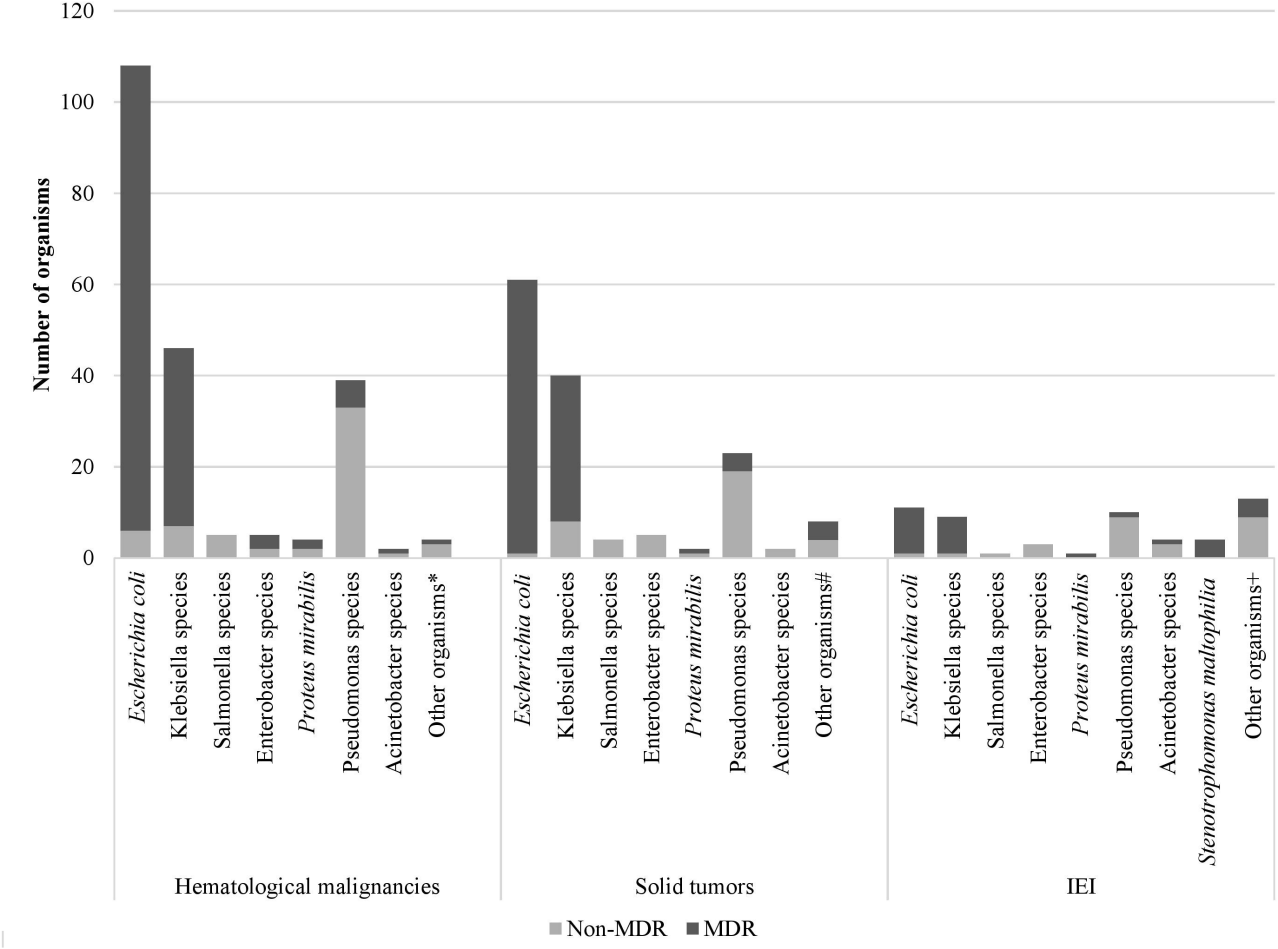
Distribution of Gram-negative bacterial isolates and their resistance phenotype according to the immunocompromised state. *Aeromonas sobria, Citrobacter koseri, Moraxella catarrhalis, Shigella flexneri # *Citrobacter freundii*, *Moraxella catarrhalis, Morganella morganii, Ralstonia piketti, Serratia liquefaciens*, *Serratia marcescens, Shigella sonnei ^+^Aeromonas hydrophilia, Brevundimonas vesicularis, Campylobacter* spp., *Chryseomonas indologens, Citrobacter freundii*, *Comamonas acidovorans, Haemophilus influenzae* not type B, *Myroides* spp., *Ochobactrum anthropic*, *Ralstonia piketti*, *Serratia liquefaciens*.

Over the study period, the most common isolated GNB were *E. coli* (43%), followed by *Klebsiella* spp. (23%) and *Pseudomonas* spp. (19%) (**Figure 2**; **Supplementary Figure S4**). Among MDR GNB, *Enterobacterales* were the most frequent isolated organisms (93.5%). *Acinetobacter* spp. and *S. maltophilia* accounted for 2% and 1% of all isolated organisms, respectively. The MDR phenotype was most common in *E. coli* (95.7%) and *K. pneumoniae* (82.7%), and this was statistically significant (**Figure 3**). When stratified by immunocompromised state, a similar distribution of the GNB was observed among hematological malignancies, solid tumors, and IEI (**Figure 4**).

**Figure 5:**
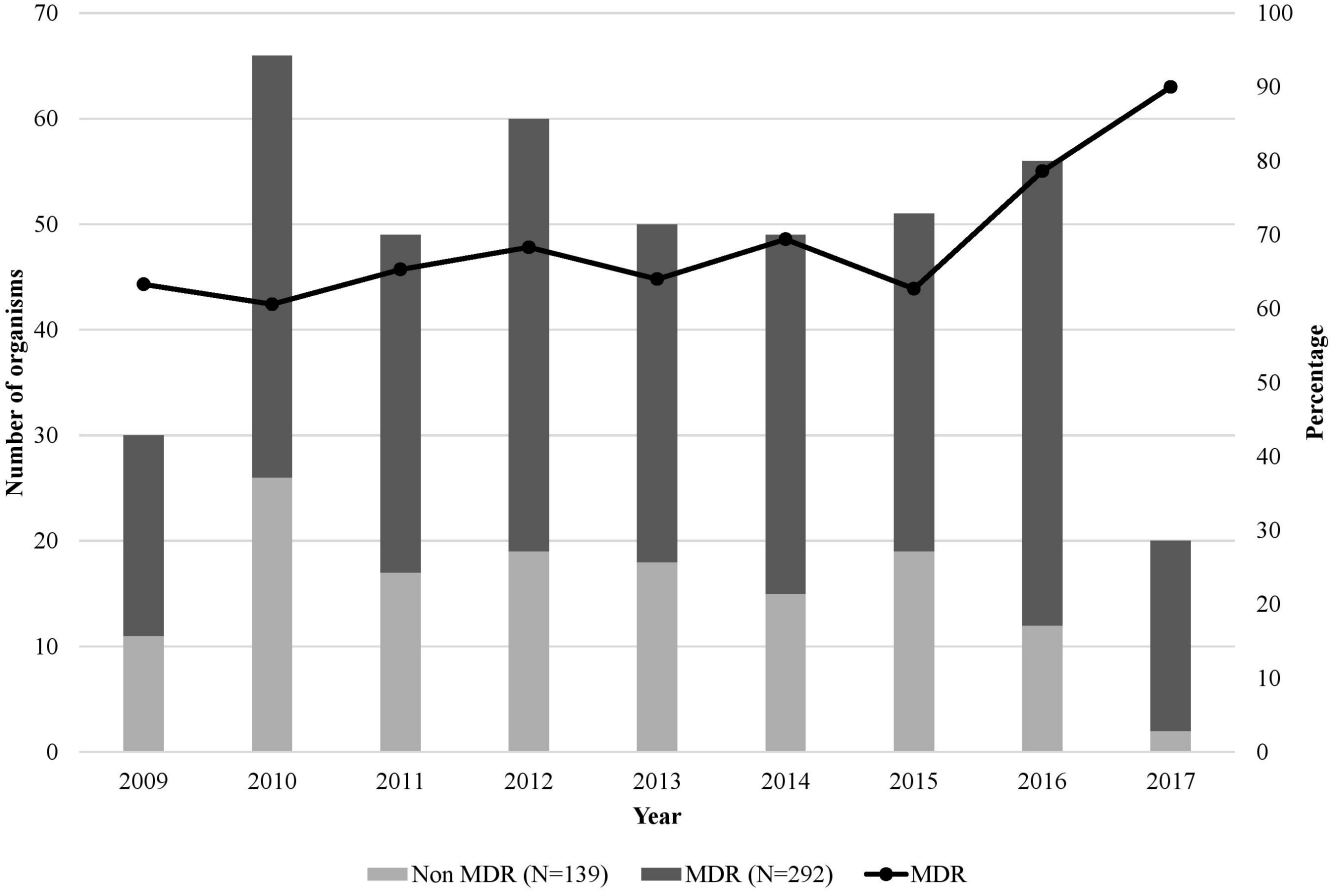
Number of Gram-negative bacterial isolates, their resistance phenotype, and percentage of multidrug resistance over the 9-year study period (N=431). The x-axis represents the years from 2009 to 2017 (to note that the included cultures were from June 1^st^ to December 31^st^ for the year 2009 and from January 1^st^ to June 31^st^ for 2017), and the y-axis is the number of organisms per year. The line graph represents the percentage of MDR GNB.

The rate of MDR GNB was almost steady from 2009 till 2015, ranging between 60% and 70%, however a sharp increase in the rate of MDR isolates was observed between 2015 and 2017, from 62.7% to 90% (**Figure 5**).

As shown in **Table 6**, 28.8% and 50.1% of isolates were from blood and urine samples, respectively. Compared to non-MDR GNB, the odds of isolating MDR GNB from urine samples were 3 times more than from blood samples.

**Table 6:** The distribution of multidrug resistant versus non-resistant Gram-negative bacterial isolates by sample source.

Sample source	Total (N=431) N (%)	Non MDR (N=139) n (%)	MDR (N=292) n (%)	OR [CI]	p-value
Blood	124 (28.8)	47 (33.8)	77 (26.5)	Ref	
Urine	216 (50.1)	36 (25.9)	179 (61.5)	**3.05 [1.83-5.08]**	**<0.001**
Skin and Soft tissue	35 (8.1)	22 (15.8)	13 (4.5)	**0.36 [0.17-0.78]**	**0.01**
Deep tracheal aspirate or sputum	18 (4.1)	12 (8.6)	6 (2.1)	**0.305 [0.11-0.87]**	**0.026**
Stool	16 (3.7)	10 (7.2)	6 (2.1)	0.37 [0.13-1.07]	0.067
Others*	18 (4.2)	11 (7.9)	7 (2.4)	—–	
Mixed**	4 (0.9)	1 (0.7)	3 (1.0)	—–	

*Others: ear discharge, eye discharge, vaginal discharge, catheter tip, CSF, biopsy (rectal, colon), pseudocyst aspirate, abdominal fluid.

**2 organisms were isolated from blood and urine, and 2 organisms from blood and skin ulcer. The bold values refer to the significant factors that have a p-value <0.05.

The antimicrobial resistance profiles of *Enterobacterales*, *Pseudomonas* and *Acinetobacter* spp. are shown in **Table 7**. Overall, *in vitro* resistance rates of *Enterobacterales* were 72.2% to third generation cephalosporins, 51.3% to fourth generation cephalosporins, 2.5% to amikacin, 38.9% to gentamicin and 32.4% to piperacillin-tazobactam. Resistance to fluoroquinolones and folate pathway inhibitors were 54% and 63.7%, respectively. *Enterobacterales* showed *in vitro* resistance to carbapenems in 7.1%. The analysis of the antimicrobial resistance profiles of *E. coli* and *K. pneumoniae* separately, showed almost similar results. Moreover, the resistance pattern to aminoglycosides, carbapenems, fluoroquinolones among *Enterobacterales* remained unchanged throughout the years. **Supplementary Figure S5** illustrates the resistance patterns among *Enterobacterales* to the antimicrobial categories that showed significant variations over the 9-year study period. There was a statistically significant increase in resistance to 4^th^ generation cephalosporins noted in 2011 when compared to 2009 (OR = 8 [2.25-28.47], p-value 0.001). This increase coincided with a decline in the use of this antimicrobial category noticed from 2011 onwards, alongside a marked increase in use of carbapenems during this period (**Supplementary Figure S3**). Our results also showed that of the 75 *Pseudomonas* spp. isolated, 11 (14.6%) were MDR. Ceftazidime and fourth generation cephalosporins resistance rate accounted for 13.5% and 12.3% in *Pseudomonas* spp. The frequency of carbapenem resistance was 17.3% (**Table 7**).

**Table 7:** Resistance to antimicrobial agents among select Gram-negative bacteria (*Enterobacterales*, *Pseudomonas* and *Acinetobacter* spp.) with focus on the multidrug resistant phenotype.

Antimicrobial Category	*Enterobacterales* (N=327)		*Pseudomonas* spp. (N=75)		*Acinetobacter* spp. (N=10)	
	Total (N=327)	MDR (N=273, 83.5%)	Total (N=75)	MDR (N=11, 14.6%)	Total (N=10)	MDR (N=3, 30%)
**Aminoglycosides**	135/314 (43.1)	134/273 (49.1)	8/75 (10.7)	8/11 (72.7)	3/10 (30.0)	3/3 (100)
Amikacin	8/314 (2.5)	8/273 (3.0)	8/75 (10.6)	8/11 (72.7)	3/10 (30.0)	3/3 (100)
Gentamicin	121/311 (38.9)	120/271 (44.3)	7/75 (9.3)	7/11 (63.6)	3/10 (30.0)	3/3 (100)
**Monobactams**	214/313 (68.4)	214/273 (78.4)	14/75 (18.7)	9/11 (81.8)	3/6 (50.0)	1/1 (100)
**Antipseudomonal penicillins+ β-lactamase inhibitors**	101/312 (32.4)	100/272 (36.8)	13/75 (17.3)	10/11 (90.9)	3/10 (30.0)	3/3 (100)
**Carbapenems**	22/313 (7.0)	22/273 (8.1)	13/75 (17.3)	9/11 (81.8)	3/10 (30.0)	3/3 (100)
**1st generation Cephalosporins**	49/56 (87.5)	48/51 (94.1)	NT	NT	NT	
**Cephamycins**	84/298 (28.2)	82/268 (30.6)	NT	NT	NT	
**2nd generation Cephalosporins**	248/310 (80.0)	246/271 (90.8)	NT	NT	NT	
**3rd generation Cephalosporins**	236/327 (72.2)	234/273 (85.7)	10/74 (13.5)	10/11 (90.4)	3/10 (30.0)	3/3 (100)
**4th generation cephalosporins**	160/312 (51.3)	160/272 (58.8)	9/73 (12.3)	8/9 (88.9)	3/10 (30.0)	3/3 (100)
**Fluoroquinolones**	176/326 (54.0)	172/273 (63.0)	8/74 (10.8)	8/11 (72.7)	3/10 (30.0)	3/3 (100)
**Penicillins**	193/208 (92.8)	182/183 (99.5)	NT	NT	NT	
**Penicillins+ β-lactamase inhibitors**	188/295 (63.7)	185/266 (69.5)	NT	NT	NT	
**Folate pathway inhibitors**	274/322 (85.1)	254/270 (94.1)	NT	NT	4/10 (40.0)	3/3 (100)
**Tetracyclins**	137/183 (75.0)	135/167 (80.8)	NT	NT	3/4 (75)	2/2 (100)
**Glycyclines**	1/58 (1.7)	1/53 (1.9)	NT	NT	2/6 (33.3)	2/3 (66.7)
**Phosphonic acids**	6/152 (3.9)	6/140 (4.3)	NT	NT	NT	
**Polymyxins**	2/8 (25.0)	2/8 (25.0)	0/6 (0.0)	0/5 (0.0)	0/5 (0.0)	0/3 (0.0)
**Nitrofurantoins**	45/177 (25.4)	40/167 (24.0)	NT	NT	NT	

Data are number of non-susceptible isolates to the antimicrobial agent/Total number of isolates tested (%)

NT, Not Tested

Species with intrinsic resistance to antimicrobial agents were removed from the analysis as indicated below:

−1^st^ generation cephalosporins: *Enterobacter cloacae, Enterobacter aerogenes, Morganella morganii, Serratia marcescens, Citrobacter freundii*.

−*2^nd^ generation Cephalosporins: Morganella morganii, Serratia marcescens*.

−Cephamycins: *Enterobacter cloacae, Enterobacter aerogenes, Citrobacter freundii*.

−*Penicillins: Enterobacter cloacae, Enterobacter aerogenes, Morganella morganii, Serratia marcescens, Citrobacter freundii, Citrobacter koseri, klebsiella* spp.

−*Penicillins + β-lactamase inhibitors: Enterobacter cloacae, Enterobacter aerogenes, Morganella morganii, Serratia marcescens, Citrobacter freundii*.

−*Glycyclines: Proteus mirabilis*.

−Tetracyclines: *Morganella morganii, Proteus mirabilis*.

*Enterobacterales* refer to: Escherichia coli (*185), Shigella spp. [Shigella flexneri (*2), Shigella sonnei (*1)]*, Klebsiella* spp. [*Klebsiella oxytoca* (*3), *Klebsiella pneumoniae* (95)], Proteus mirabilis (*8), *Citrobacter* spp. [Citrobacter koseri (*1), Citrobacter freundii (*3)], *Serratia* spp. [Serratia marcescens (*1), Serratia liquefaciens (*2)], Morganella morganii (*2), *Enterobacter* spp. [*Enterobacter aerogenes* (*1), *Enterobacter cloacae* (*11), *Enterobacter agglomerans* (*1)], *Salmonella* spp. [*Salmonella enteritidis* (*7), *Salmonella* group C (*2), *Salmonella paratyphi* (*1), *Salmonella* not typable (*1)].

*Pseudomonas* spp. refer to: *Pseudomonas aeruginosa* (*64), *Pseudomonas fluorescens* (*3), *Pseudomonas putrefaciens* (*1), *Pseudomonas stutzeri* (*6), *Pseudomonas* spp. (*1).

*Acinetobacter* spp. refer to: *Acinetobacter anitratus* (*4), *Acinetobacter baumanii* (*5), *Acinetobacter lwoffi* (*1). The bold values refer to the significant factors that have a p-value <0.05.

The percentages of resistance observed in *Acinetobacter* spp. against the tested antimicrobial categories were 30% to each 4^th^ generation cephalosporins, carbapenems, aminoglycosides and fluoroquinolones. In addition, the 3 MDR *Acinetobacter* isolates were resistant to all tested antimicrobial agents except colistin, and glycyclines (**Table 7**). The resistance pattern to the different antimicrobial agents among *Pseudomonas and Acinetobacter* spp. did not show a statistically significant change over the study period, except for resistance to the folate pathway inhibitors among the *Acinetobacter* spp. All 4 isolated *Acinetobacter* spp. isolated in 2010 and 2016 were 100% resistant to the folate pathway inhibitors, whereas those isolated in 2011, 2013 and 2014 (n=6) were all susceptible to this antimicrobial category.

## Discussion

MDR GNB are a significant threat in immunocompromised patients, who are at high risk of overwhelming infections, in addition to complicating treatment and increasing mortality and morbidity (Costa et al., 2015; Mechergui et al., 2019). Hence, familiarity with local epidemiology and resistance patterns is crucial to initiate the appropriate treatment without any delay. Data regarding the epidemiology of infections with MDR GNB in pediatric immunocompromised patients in Lebanon and the MENA region are scarce and therefore this study aimed to describe the frequency of MDR GNB infections, associated risk factors as well as the resistance profiles over a 9-year period.

The widespread use of empirical antibiotic therapy and prophylaxis with agents that have broad activity against GNB, especially during episodes of febrile neutropenia, has contributed to the increase in the rate of MDR isolates (Hernández-Jiménez et al., 2022). In this study, 72% of the isolates were considered MDR. This finding is compatible with other regional studies conducted in Egypt by Tohamy et al. and El-Mahallawy et al. that reported MDR was identified in almost 69% of bloodstream infections in pediatric and adult cancer patients (El-Mahallawy et al., 2014; Tohamy et al., 2018). According to the 2023 updated guidelines for the management of fever and neutropenia in pediatric patients with cancer and hematopoietic cell transplantation recipients, monotherapy with an antipseudomonal β-lactam, a fourth-generation cephalosporin or a carbapenem are recommended as empiric antibacterial therapy in pediatric high-risk (Lehrnbecher et al., 2023).

The mean age in our retrospective study was 7.7 years and most patients had hematological malignancies followed by solid tumors. Similarly, a systematic review by Haeusler et al., including 5 cohort studies on the associated factors with resistant GNB in children with cancer, showed that the median age of children was between 5 and 6 years and the majority had a hematological malignancy (Haeusler and Levene, 2015). In line with the finding of our present study, a prospective study at a single center in Thailand showed that females were an independent risk factor for MDR hospital infections (Sritippayawan et al., 2009). On the contrary, an observational prospective multicentered study conducted on patients less than 18 years of age in Hungary, over a 2 year period showed that male gender was associated with higher rate of MDR bloodstream infections (39.8% vs. 23.5% in females) on multivariate analysis (Ivády et al., 2016). The gender difference is probably due to the inclusion of isolates from patients with UTI, such as in our study, where there’s a clear female predominance.

Our study revealed that chemotherapy and previous infection or colonization with the same organism were independent risk factors for infections with MDR GNB. These results are consistent with the study findings of Haeusler et al. who reported that high intensity chemotherapy and isolation of antibiotic resistant GNB from any site within the preceding 12 months are independent risk factors associated with antibiotic resistant bacteremia in pediatric oncology patients (Haeusler et al., 2013). Moreover, Aizawa et al. showed, in their retrospective study conducted between April 2010 and March 2017 at 8 Japanese children’s hospitals, that MDR bloodstream infections were independently associated with cancer chemotherapy within 30 days (Aizawa et al., 2019).

Neutropenia and antibiotic use in the previous 30 days were identified as significant risk factors for infections with MDR GNB in our study, and these findings were compatible with other studies (Zaoutis et al., 2005; El-Mahallawy et al., 2011b). The selective pressure exerted by antimicrobial exposure plays a major role in the development of MDR organisms (Haeusler et al., 2013; Averbuch et al., 2017). Furthermore, the administration of broad-spectrum antibiotics during episodes of febrile neutropenia or other indications and prolonged antibiotic prophylaxis are well-established factors contributing to the colonization with MDR GNB (Hernández-Jiménez et al., 2022). Thus, it is essential to limit antibiotic exposure through early discontinuation of antibiotic therapy when appropriate and de-escalation to a narrower spectrum treatment with the intention to decrease antibiotic pressure and to prevent further development of resistance (Averbuch et al., 2017).

We found that the most frequent microorganisms isolated among GNB were *E. coli* (43%), followed by *Klebsiella* (23%) and *Pseudomonas* spp. (17%). These data are consistent with findings from other studies (Trecarichi et al., 2015b; Tripathi et al., 2023). The majority of the MDR isolates were from urine (61.5%), followed by blood (26.5%) and the remainder from respiratory, skin, or surgical site and others. This is similar to the findings by Uzodi et al. where the majority of the MDR isolates were from urine (83%) (Uzodi et al., 2017),. Among MDR GNB, *Enterobacterales* were the most frequent isolated organisms (93.5%). It is noteworthy that our study found alarming rates of MDR among *E. coli (95.7%) and Klebsiella* spp. (82.7%), similarly to a study by Kamel et al. at a children’s cancer hospital in Cairo, Egypt during the period of October 2014 to December 2016 (Kamel et al., 2018).

In our study, 7.1% of *Enterobacterales* were carbapenem resistant. In Lebanon, at our center, a notable increase was observed in the percentages of CR, from 2010 to 2016, for *E. coli* (0.1% to 5%) and *K. pneumoniae* (0.7% to 8%) (Araj et al., 2018). In fact, it is crucial to highlight the increasing rate of MDR pathogens and the broad dissemination of ESBL and carbapenemase-producing GNB in the Middle Eastern countries, due to multiple factors including inappropriate use of antibiotics and substantial population migration (Al Dabbagh et al., 2023). Among the numerous challenges posed by MDR GNB, the emergence and spread of carbapenem resistance represents the most urgent concern, given the limited availability of alternative treatments that are both effective and safe and the near-empty pipeline for new effective antibiotics, especially in the pediatric age group (Chiotos et al., 2015; Aguilera-Alonso et al., 2020). Our study showed an increase in resistance to 4^th^ generation cephalosporins among *Enterobacterales*, mainly in 2011 coinciding with a decline in the use of this antimicrobial category and an increase in theuse of carbapenems. Consequently, this can explain the later decrease in resistance to 4^th^ generation cephalosporins among *Enterobacterales*, followed by a subsequent increase in the use of this category in 2015. Additionally, the aminoglycoside use started to increase in 2012, likely related to its concurrent administration with carbapenems or cephalosporins as part of a combination antimicrobial therapy. Moreover, we observed a worrisome resistance rate to fluoroquinolones (54%) among *Enterobacterales*, similar to what has been reported in recent epidemiologic studies which highlighted that their extensive use potentially contributed to the decline in susceptibility to quinolones (Jacobson et al., 1999; Mihu et al., 2010; Bhusal et al., 2011). Fortunately, these management changes and the institution of an antimicrobial stewardship program at our medical center resulted in a gradual decrease in the IRR in immunocompromised patients over the course of the study despite the alarming increase in AMR in GNB (**Supplementary Figures S1**, **S2**).

*Pseudomonas* spp. are an invasive GNB responsible for severe invasive diseases. The antibiotic resistance via overexpression of efflux pumps, decreased expression of porin, mutations in quinolones targets and the low permeability of the cell wall, make *Pseudomonas* spp. highly susceptible to become MDR (Caselli et al., 2010). Our study revealed that 14% of *Pseudomonas* spp. were MDR. In addition, 17.3% and 25% were resistant to antipseudomonal penicillins and antipseudomonal cephalosporins, respectively. A retrospective study conducted between 2000 and 2008 in the pediatric hematology oncology Italian network, reported that 31.4% of *Pseudomonas aeruginosa* isolates were MDR, 27% and 33% were resistant to antipseudomonal penicillins and cephalosporins, respectively (Caselli et al., 2010). Furthermore, resistance to amikacin and fluoroquinolones in our study was identified in almost 11% of the *Pseudomonas spp*, and this was compatible with the study by Caselli et al (Caselli et al., 2010).

*Acinetobacter baumannii* is an important Gram-negative coccobacillus opportunistic organism with a remarkable ability to acquire antibiotic resistance that can cause severe infections in patients with immune dysfunction (Shi et al., 2020). In the current study, we reported that 30% of *Acinetobacter spp* were MDR (3 out of 10 isolates). According to the standard definitions for acquired resistance (Magiorakos et al., 2012), 2 of these 3 resistant isolates can be classified as extensively drug-resistant (XDR) as these isolates showed non-susceptibility to at least one agent in all but two or fewer antimicrobial categories. All the MDR *Acinetobacter spp* were resistant to aminoglycosides, carbapenems, cephalosporins and folate pathway inhibitors. Similar findings were reported by Shi et al. at the PICU of Shanghai Children’s Hospital in China from December 2014 to May 2018, and by Balkhy et al. between October 2001 and December 2007 at King Abdulaziz Medical City in Riyadh, Saudi Arabia (Shi et al., 2020).

Due to its retrospective design, this study has several limitations including missing data and incomplete medical records. The single-center nature of this study limits the generalizability of the results. Therefore, future national multicenter studies should be conducted among patients with gram-negative infections to determine the local prevalence of MDR and identify the possible risk factors. Such studies will offer valuable guidance to develop effective interventions such as antimicrobial stewardship programs, to stabilize or reduce the spread of multidrug resistance and mitigate the morbidity and mortality associated with the widespread MDR GNB infections. To the best of our knowledge, this is one of the first pediatric studies on the epidemiology of MDR GNB in immunocompromised patients, in addition to being a 9-year study.

The strengths of this paper reflect the Antimicrobial Stewardship Program (ASP) at AUBMC. The antimicrobial stewardship remains at the core of efforts to reduce the burden of infections with MDR GNB. The World Health Organization issued guidance in 2019 on how to establish an ASP in low- and middle-income countries (WHO, 2019). The Lebanese Ministry of Public Health called for the establishment of ASP at all Lebanese hospitals in response (Health MOP, 2019). The AUBMC had already launched its official ASP in 2017, in compliance with the Joint Commission International Standards, with a full time dedicated clinical pharmacist and a director of the program (AUB, 2024). These efforts aim to limit inappropriate use of antibiotics, stabilize, or decrease antibiotic resistance and to improve patient outcomes. Thus, enhanced surveillance and data sharing are needed to fully describe the antimicrobial resistance rates, especially after the implementation of antibiotic stewardship interventions in some hospitals.

## Conclusion

Infections with MDR GNB are a significant evolving threat to all patients in general, and to immunocompromised children in particular. The findings of our study shed light on the importance and the urgent need for sustainable bacterial surveillance, antimicrobial stewardship programs, and effective infection control measures, notably during this critical period of economic collapse resulting in drug shortages and other challenges in this country and other war-inflicted countries.

## Data Availability

The datasets presented in this article are not readily available because data is available upon request due to privacy and ethical restrictions. Requests to access the datasets should be directed to gdbaibo@aub.edu.lb.
