# Supplementary material for: Increased rate of multidrug-resistant gram-negative bacterial infections in hospitalized immunocompromised pediatric patients
Source: Front Cell Infect Microbiol. 2025 Jan 6;14:1382500. doi: 10.3389/fcimb.2024.1382500 (PMC11743943; doi:10.3389/fcimb.2024.1382500)
Supplement: Supplementary file 1 [file DataSheet1.docx]

**Supplementary material**

**Figure 1S. The incidence of MDR GNB per 100,000 immunocompromised patients per year from 2009 to 2017.**

**Figure 2S. The incidence rate ratios (IRRs) of MDR GNB among immunocompromised pediatric patients across years.**

The IRR was calculated by dividing the incidence rate of the current year by that of the previous year.

**Figure 3S. The different antimicrobial categories use for the treatment of MDR GNB infections over the 9-year study period.**

The x-axis represents the years from 2009 to 2017 (to note that the included cultures were from June 1^st^ to December 31^st^ for the year 2009 and from January 1^st^ to June 31^st^ for 2017), and the y-axis is the percentage of antimicrobial category use per year.

**Figure 4S. Gram-negative bacterial organisms’ distribution over the 9-year study period.**

The x-axis represents the years from 2009 to 2017 (to note that the included cultures were from June 1^st^ to December 31^st^ for the year 2009 and from January 1^st^ to June 31^st^ for 2017), and the y-axis is the number of organisms per year.

*Organisms: *Aeromonas hydrophilia*, *Aeromonas sobria*, *Brevundimonas vesicularis*, *Campylobacter* species (2), *Chryseomonas indologens* (2), *Citrobacter freundii* (3), *Citrobacter koseri*, *Comamonas acidovorans*, *Haemophilus influenzae* not type B, *Moraxella catarrhalis* (2), *Morganella morganii* (2), *Myroides* species, *Ochobactrum anthropi*, *Ralstonia piketti* (2), *Serratia liquefaciens* (2), *Serratia marcescens*, *Shigella flexneri* (2), *Shigella sonnei*.

**Figure 5S. The resistance rate to some antimicrobial categories among *Enterobacterales* over the 9-year study period.**

The x-axis represents the years from 2009 to 2017 (to note that the included cultures were from June 1^st^ to December 31^st^ for the year 2009 and from January 1^st^ to June 31^st^ for 2017), and the y-axis is the resistance rate to the antimicrobial category (%) per year.

Only the antimicrobial categories showing significant variation over the study period are represented in this figure.
